# Validation of accuracy deformable image registration contour propagation using a benchmark virtual HN phantom dataset

**DOI:** 10.1002/acm2.13246

**Published:** 2021-05-04

**Authors:** Robert Boyd, Amar Basavatia, Wolfgang A. Tomé

**Affiliations:** ^1^ Department of Radiation Oncology Montefiore Medical Center Bronx NY USA; ^2^ Institute for Onco‐Physics Albert Einstein College of Medicine Bronx NY USA

**Keywords:** deformable registration, kVCT, MVCT, registration accuracy, virtual phantoms

## Abstract

Virtual anatomic phantoms offer precise voxel mapping of the variation of anatomy with ground truth deformation vector fields (DVFs). Dice similarity coefficient (DSC) and mean distance to agreement (MDA) are the standard metrics for evaluating geometric contour congruence when testing deformable registration (DIR) algorithms. A HN virtual patient phantom data set was used for a kVCT‐kVCT automatic propagation contour validation study employing the Accuray DIR algorithm. Furthermore, since TomoTherapy uses MVCT images of the relevant anatomy for adaptive monitoring, the kVCT image data set quality was transformed to an MVCT image data set quality to study intermodal kVCT‐MVCT DIR accuracy. The results of the study indicate that the Accuray DIR algorithm can be expected to autopropagate HN contours adequately, on average, within tolerances recommended by TG‐132 (DSC 0.8‐0.9, MDA within voxel width). However, contours critical to dosimetric planning should always be visually proofed for accuracy. Using standard reconstruction MVCT image quality causes slightly less, but acceptable, agreement with ground truth contours.

## 1 INTRODUCTION

Deformable image registration (DIR) algorithms, widely available in commercial software, have come into regular use in the clinic for tasks such as autosegmentation and adaptive planning. AAPM Task Group 132 provides recommendations for quality assurance and quality control of clinical processes involving both rigid and deformable registration.^1^ The TG‐132 dataset, while suitable for general commissioning and quality assurance (QA) of DIR software, is limited to in‐depth, site‐specific validation of DIR algorithms due to unique anatomic variations, both spatial and temporal, associated with a given treatment site. TG‐132 recommends the use of 10 patient datasets to evaluate site‐specific registration methods. Frederick et al.^2^ proposed a standardized framework for validating automatic contour propagation using interobserver expert contouring on both the primary and secondary patient image sets. Congruence of DIR‐propagated contours and expert contours can be evaluated geometrically or dosimetrically; the geometric congruence metrics recommended by TG‐132 are as follows: (a) mean distance to agreement (MDA) and (b) Dice similarity coefficient (DSC).

As an alternative, virtual anatomic phantoms can provide precise voxel mapping of the anatomic variation with ground‐truth deformation vector fields (DVFs) for the evaluation of DIR algorithms.^3^, ^4^, ^5^, ^6^, ^7^, ^8^, ^9^, ^10^, ^11^ In addition to geometric congruence, the distribution of the voxel registration error for a specified target volume (TRE) can be a useful metric for comparative DVF analysis.^9^

A library of 10 HN interfraction virtual anatomic phantoms from patient CT image data is available for evaluating DIR algorithms.^11^ Each test case in the library consists of a start‐of‐treatment (SOT) and a simulated end‐of‐treatment (sEOT) kVCT image set. These sEOT images sets were generated by deforming the SOT image sets to match the actual EOT image sets. As recommended by TG‐132, DVFs from two different deformation models (biomechanical and thin‐plate) were used sequentially and combined to minimize biased validation results. Patients selected for the library lost 4‐20% of body weight (average10.6%) from SOT to EOT. The two deformation models allowed Pakula and colleagues^9^ to model head/mandible rotations and translations, spine flexion, shoulder position, hyoid movement, tumor/node shrinkage, weight loss, and parotid shrinkage. The entire HN dataset, including SOT contours and DVFs, is available through Oncology Systems Limited. The SOT kVCT (kVCT_SOT_) and simulated EOT kVCT (kVCT_sEOT_) are available for download from the Deformable Image Registration Evaluation Project (DIREP) website. Pukala et al.^9^ used the HN dataset to benchmark dose accumulation congruence metrics for five commercial DIR algorithms; however, the Accuray DIR algorithm was not part of their study.

The Accuray DIR model (Accuray Inc., Sunnyvale, CA) uses a non‐parametric, non‐rigid transformation to represent the deformation field.^12^ The algorithm optimizes the normalized cross‐correlation (NCC) over small neighborhood patches. The optimization occurs iteratively over the entire image domain in a multi‐resolution, coarse‐to‐fine scheme, using up to 4 resolutions and up to 500 iterations at each resolution level. The estimated deformation field is regularized after each iteration with a smoothing operator. The Accuray Precision treatment planning system utilizes this DIR algorithm for atlas‐based autosegmentation, re‐planning contour propagation, adaptive therapy dose accumulation, and MVCT dose‐of‐the‐day metrics.

Deformable registration works best with feature‐rich images with high contrast variations.^13^ However, the lower image contrast of MVCT images can limit registration accuracy.^14^ Noise degrades contrast and DIR accuracy,^15^ and TG‐132 recommends adding noise to virtual phantoms to better model imaging systems. Several studies added CBCT quality noise to kVCT patient image data to evaluate the impact of kVCT‐CBCT intermodal DIR.^7^, ^16^

The purpose of this study was to present a validation of Accuray DIR automatic contour propagation using a publicly available HN dataset, and, hence, a methodology using these publicly available HN datasets for contour propagation studies is presented for further comparative studies. Furthermore, to validate the clinical use case in which kVCT images are deformably registered to MVCT images, the HN kVCT_sEOT_ image data sets were transformed to MVCT image data sets to evaluate the impact of MVCT image quality on DIR.

## 2 MATERIALS AND METHODS

Table 1 lists the HN contours of interest for the study along with the average and standard deviation (SD) volume and the number of contour sets used for each contour of interest. Some contour sets were missing from the dataset or were rejected based on an inverse consistency threshold discussed below. The CT_SOT_ contours were propagated to the CT_sEOT_ image set for each HN test case using inverted ground‐truth DVFs to compare with auto‐propagated Accuray DIR contours. The CT_SOT_ contours supplied with the HN dataset were not modified.

**Table 1:** List of HN contours of interest sets used for validation study along with average volume (±SD).

Contour		Volume (cc) Ave (±SD)
Mandible	*n* = 9	71.8 ± 16.0
Oral cavity	*n* = 7	31.3 ± 14.9
Brainstem	*n* = 9	29.7 ± 7.4
Spinal cord	*n* = 9	22.8 ± 7.5
Parotid gland	*n* = 18	19.9 ± 7.7
Pharyngeal constrictors	*n* = 6	11.1 ± 4.7
Esophagus	*n* = 9	6.6 ± 2.5
Submandibular gland	*n* = 15	5.3 ± 2.4
Larynx	*n* = 9	5.1 ± 2.0

### 2.A Generation of HN dataset CTsEOT contours

Only the CT_SOT_ contours are available in the HN dataset. For the purpose of validating contour propagation, DVFs available in the HN dataset were used to generate the CT_sEOT_ contours. The DVFs are inversions of the ground‐truth DFVs used to generate the CT_sEOT_ image data from the CT_SOT_ image data. They had been inverted to analyze CT_sEOT_ to CT_SOT_ deformation mapping for reference dose accumulation.^9^ ImSimQA version 4.2 (Oncology Systems Limited, Shrewsbury, UK) was used to “re‐invert” the DVFs (back to their original source–target direction) to propagate the SOT contours to the CT_sEOT_ image set. The DVF inversion was validated using the ImSimQA inverse consistency error (ICE) tool. Similar to using the ImSimQA DVF comparison tool to analyze the TRE distribution, contours were selected for the volumetric analysis of the voxel ICE distribution.

For the nine of the test sets, 79% of the contours of interest had mean ICE distances of <0.005 mm, and 97% had mean ICE distances of <0.05 mm. However, three contours had mean ICE distances of >0.2 mm and were excluded from the study. All the contours of interest in test case 7 had mean ICE distances > 0.5 mm, and for this reason, this test case has been excluded from the study.

### 2.B Accuray DIR contour propagation

The Accuray DIR algorithm is an automated, proprietary process that is integrated into a MIM viewer with limited options for the user. Contours from both the initial rigid registration and the subsequent deformable registration are available in the MIM viewer for further analysis and modification. The Precision Re‐Treatment module allows for the verification and manual fine‐tuning of the initial rigid alignment before deformable registration is applied. Using the TG‐132 translation geometric phantom in the Precision Retreatment module, we verified the initial alignment for all three noncoplanar 2‐mm spherical markers was better than 0.1 mm in all directions.

A simple plan in Precision was calculated for each test case using the CT_SOT_ image set and SOT contours of interest to allow the use of the Precision Re‐Treatment module. The module was used to automatically apply deformable registration and subsequent contour propagation to each of the EOT image sets, that is, the kVCT_s__EOT_ and simmulated MVCT_EOT_ image sets. The option to fine tune rigid registration manually before deformable registration was bypassed. Contours were exported to MIM version 6.9.4 (MIM Software Inc., Cleveland, OH) for the analysis of MDA and DSC metrics.

As an additional test, three patients who were re‐simulated midway through treatment and manually re‐contoured for adaptive treatment were selected for comparison with the results of the HN phantom dataset validation study. The same procedure as above was used to generate contours and congruence with manually drawn contours was analyzed in MIM. This clinical study was limited to kV‐kV DIR.

### 2.C MVCT image quality modeling

**Fig. 1:**
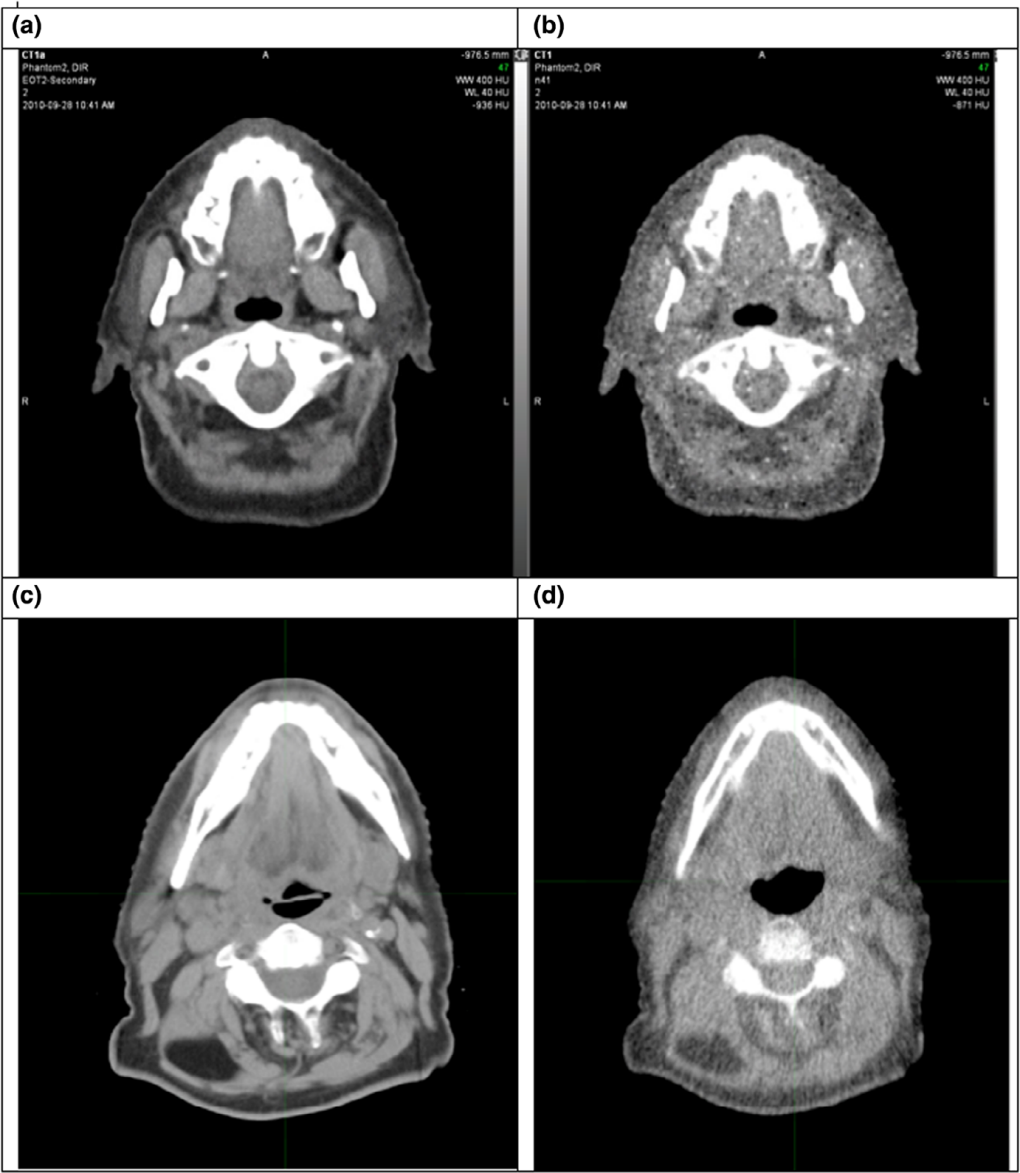
HN virtual phantom image slice before (a) and after (b) MVCT image quality transformation. An actual clinical kVCT (c) and MVCT (d) HN image slice for qualitative comparison.

The transformation of HN kVCT_sEOT_ image data to MVCT quality (MVCT_EOT_) consisted of three steps: (a) smoothing images, (b) scaling the dynamic range, and (c) adding MVCT random noise quality. Image data from kVCT and MVCT scans of a TomoTherapy “Cheese” phantom TomoTherapy were used to model MVCT image quality. The kVCT scans were performed on a Siemens MX8000 using 3 mm slice spacing, and the MVCT scans were performed on an Accuray TomoTherapy HD using coarse scanning and 3 mm slice standard reconstruction. All three steps of CT_sEOT_ image modification were accomplished using Python 3.7. Figure 1 shows a CT_sEOT_ image slice before and after MVCT image quality transformation along with an actual clinical kVCT and MVCT image slice for qualitative comparison.

**Fig. 2:**
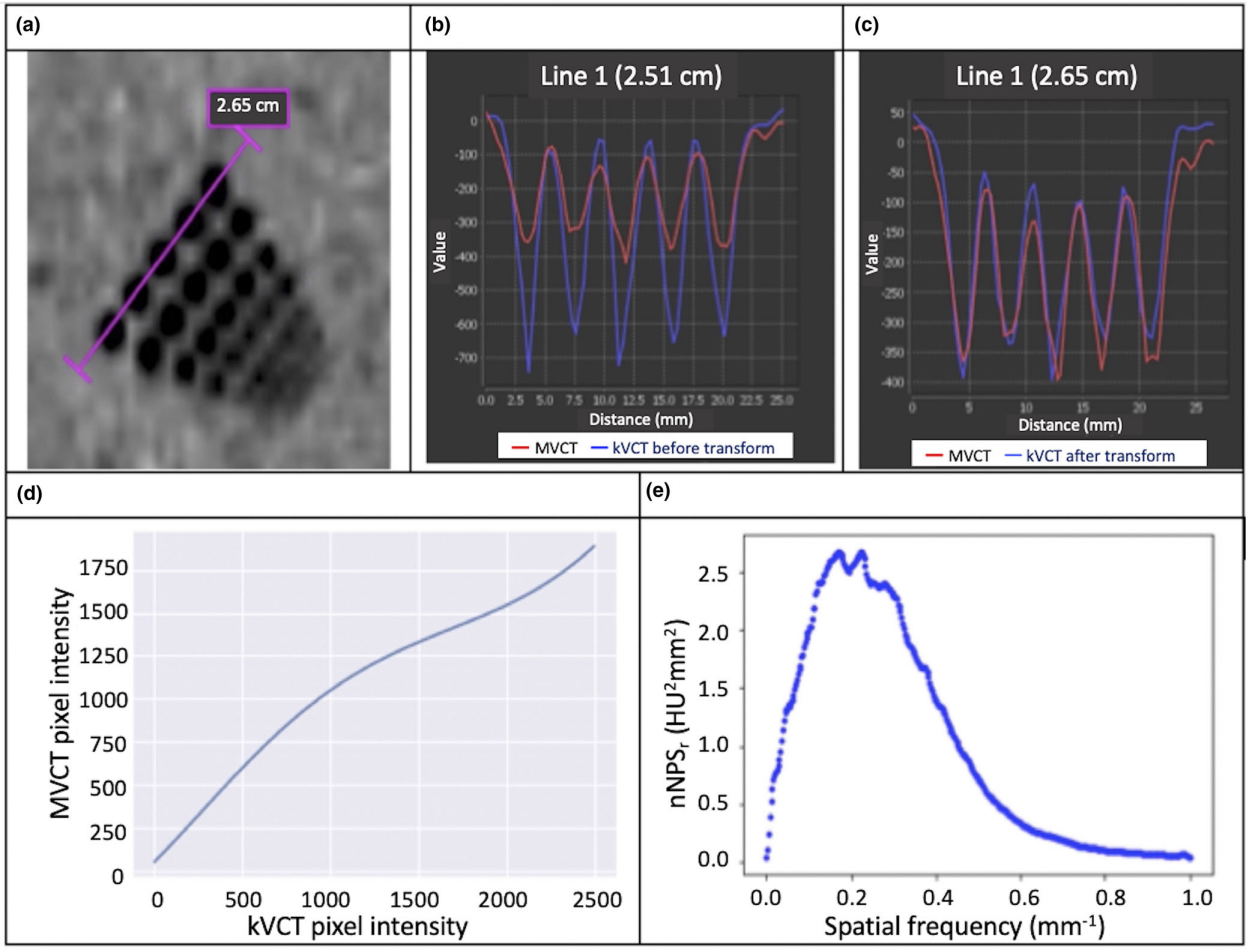
MVCT image quality transforms. (a) shows the line through resolution holes for sampling pixel intensity profiles shown in (b) and (c), where true MVCT is red and kVCT is blue [before (b) and after (c) image quality transformation]. (d) shows the kVCT‐to‐MVCT pixel intensity conversion function. (e) shows the radial profile of the measured MVCT noise power spectrum.

MVCT‐quality random noise quality was added to each modified CT_EOT_ image slice. The measured MVCT noise power spectrum (NPS) was extracted from two consecutive MVCT scans of the uniformity section of the “Cheese” phantom using the approach proposed by Friedman and colleagues [cf. Fig. 2(e)].^17^ To generate an MVCT spatial noise image quality, a Gaussian white noise (sigma = 4%) image mask (GWNIM) was generated and the positive and negative definite parts of the GWNIM were then Fourier transformed separately and folded with the NPS in frequency space. The resulting positive and negative definite parts of the MVCT spatial noise pattern in frequency space were then inversely Fourier transformed to obtain the positive and negative definitive part of the spatial MVCT spatial noise pattern and were then combined into a single MVCT quality spatial noise pattern by subtracting the negative definite part from the positive definite part. The resulting MVCT‐quality spatial noise pattern was then added to the kVCT image, and following that, a 0.5‐pixel Gaussian smoothing was applied to the MVCT noise image to slightly blur the distinct salt‐and‐pepper pattern. The resulting additive noise was +30 HU. A separate and new MVCT‐quality spatial noise pattern was generated for each CT slice in the KVCT data set.

For each test case, three associated MVCT_EOT_ image sets were created by adding a separate MVCT‐quality spatial noise map (4% spatial MVCT noise) to each KVCT image slice in the KVCT_s__EOT_ image set. For each test case, MDA and Dice metrics of the three kV‐MV DIR results were averaged. This allowed a better estimate of the expected differences between kV‐kV and kV‐MV DIR modes. The Wilcoxon rank test was used to determine if the differences between kV‐kV and kV‐MV DIR metrics were statistically significant.

## 3 RESULTS

### 3.A Accuray DIR algorithm process

**Fig. 3:**
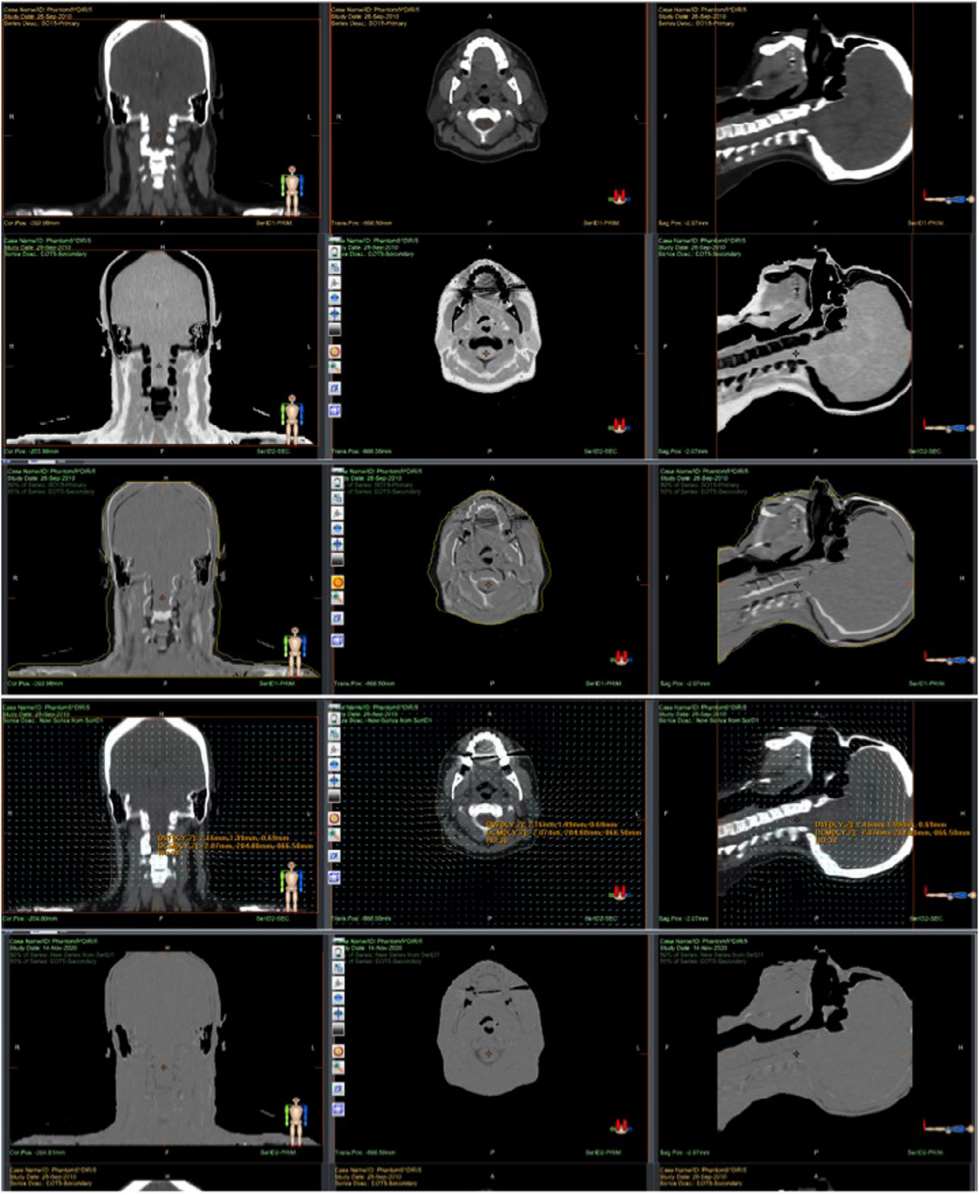
Graphical representation of Accuray DIR process. The first row shows the coronal, axial, and sagittal views of the kVCT_SOT_ image set, which is the moving image set, and the second row shows views of the kVCT_EOT_ (with inverse grayscale voxel intensity), which is the stationary image set. The third row shows the fusion of the two image sets using a 50% blend before DR, along with the body contour of the SOT image to accentuate the missing tissue from weight loss. The fourth row shows views of the resulting DR image with an overlay of the DVF vectors, while the fifth row shows the overlap of the DR SOT image set with the original SOT image set.

Figure 3 shows a graphical representation of the Accuray DIR process using phantom test case 5. By fusing the SOT image set with the EOT image set (represented by inverse grayscale voxel values) and applying a 50% blend, the differences due to weight loss and setup variation, such as head rotations, appear most notably where there is a bony mismatch. An overlay of the body contour from the SOT image set allows for better visualization of missing tissue. An overlay of the DVF vectors on the deformed SOT image shows how the algorithm accounts for weight loss, spine inflexion, and head rotations. A 50% blend of the deformed moving SOT image and the stationary EOT image (using inverse grayscale) shows a relatively uniform distribution of less intense features from intensity mismatch.

### 3.B kV‐kV contour congruence

**Fig. 4:**
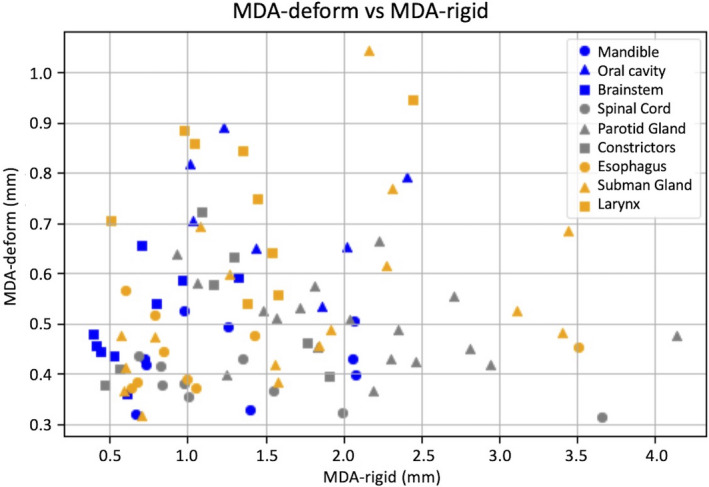
Plot of MDA_DR_ vs MDA_RR_ for all kV‐kV contour propagations. The contours are ordered in the legend by average volume from largest to smallest.

**Fig. 5:**
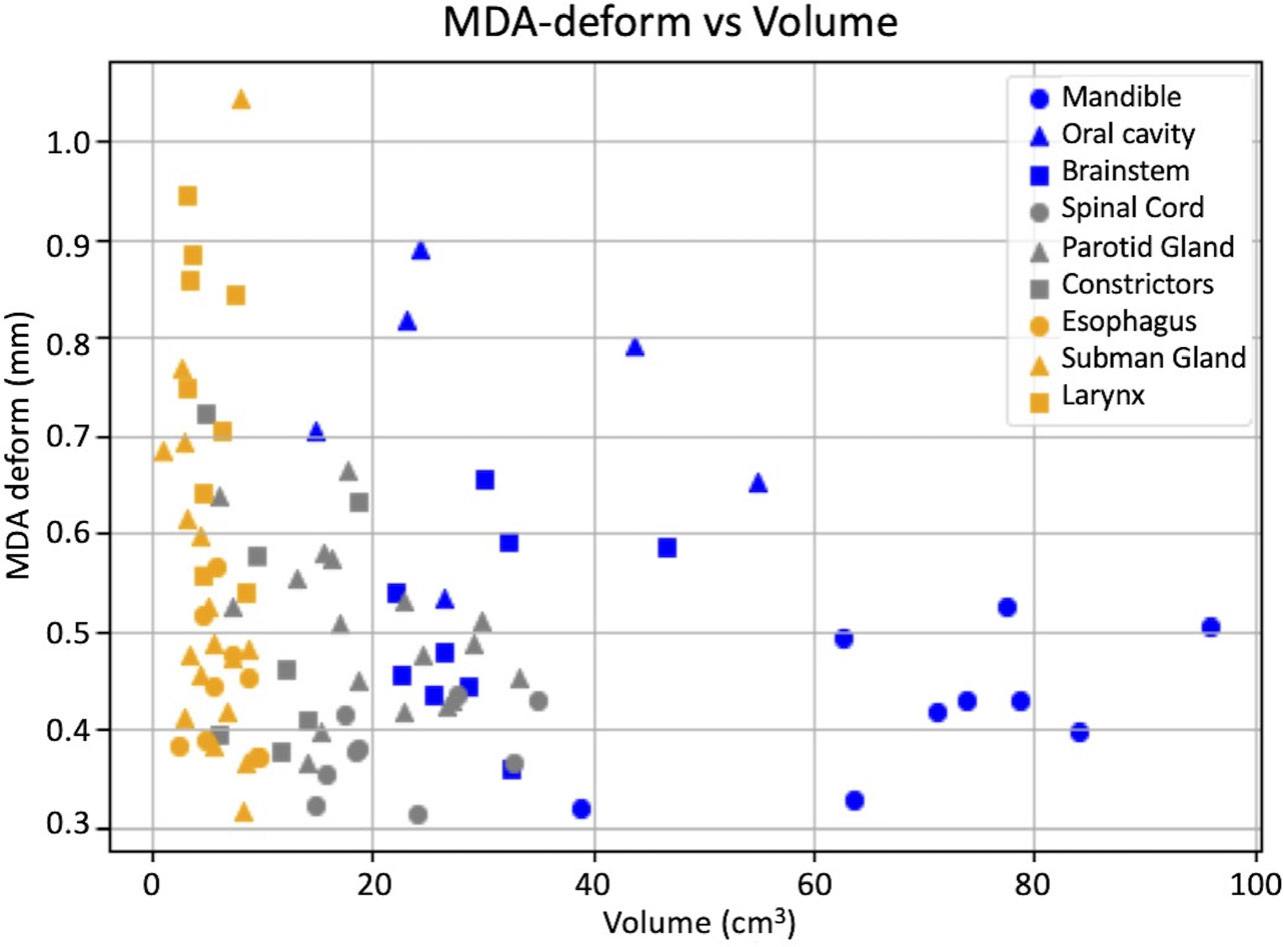
Plot of MDA_DR_ vs contour volume (cc) for all kV‐kV DR contour propagations.

The kV‐kV MDA geometric congruence in millimeters is summarized in Table 2. For each contour group, the table lists the average MDA and standard deviation (SD) for both rigid registration (RR) and deformable registration (DR) contours. On average, the MDA_DR_ distances are well within a voxel width as recommended by TG‐132. The voxel dimensions of the image data in the HN library are approximately 1 × 1 × 3 mm^3^. The MDA_RR_ distances are within 5 mm millimeters which indicates that the initial rigid alignment was acceptable (cf. Fig. 4). It is good practice to always first do a RR to bring the image sets into alignment as close as possible before proceeding with a DR. Most notable was the MDA_RR_ for the brainstem which was under 1 mm and <0.1 mm variance. Figure 4 shows the relationship between MDA_RR_ and MDA_DR_ for all contours of interest and illustrates the improvement in geometric convergence in the algorithm process step from RR to DR. Figure 5 shows the relationship between contour volume and MDA_DR_ for all contours of interest. The volume shown for each contour of interest is the average of the SOT and EOT contour volumes.

**Table 2:** Average (±SD) MDA in mm for both RR and DIR kV‐kV contour propagation.

Contour	MDA_RR_ (mm) Ave ± SD	MDA_DR_ (mm) Ave ± SD
Mandible	1.3 ± 0.6	0.4 ± 0.1
Oral cavity	1.6 ± 0.5	0.7 ± 0.1
Brainstem	0.7 ± 0.03	0.4 ± 0.1
Spinal cord	1.4 ± 0.9	0.5 ± 0.1
Parotid gland	2.1 ± 0.8	0.5 ± 0.1
Pharyngeal constrictors	1.2 ± 0.6	0.7 ± 0.2
Esophagus	1.2 ± 0.9	0.6 ± 0.2
Submandibular gland	1.7 ± 1.0	0.6 ± 0.1
Larynx	1.4 ± 0.5	0.7 ± 0.2

**Fig. 6:**
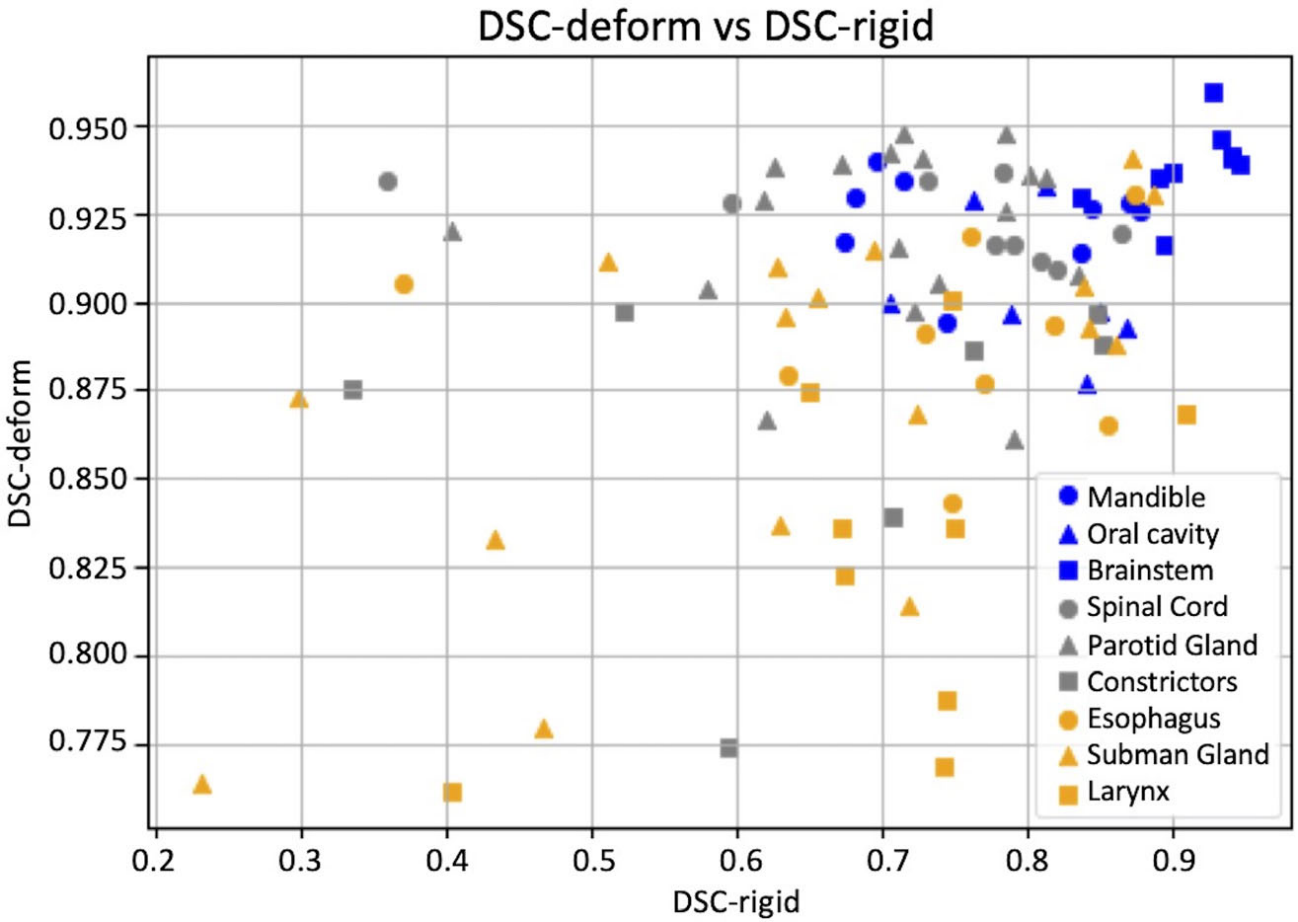
Plot of DSC_DR_ vs DSC_RR_ for all kV‐kV contour propagations.

**Fig. 7:**
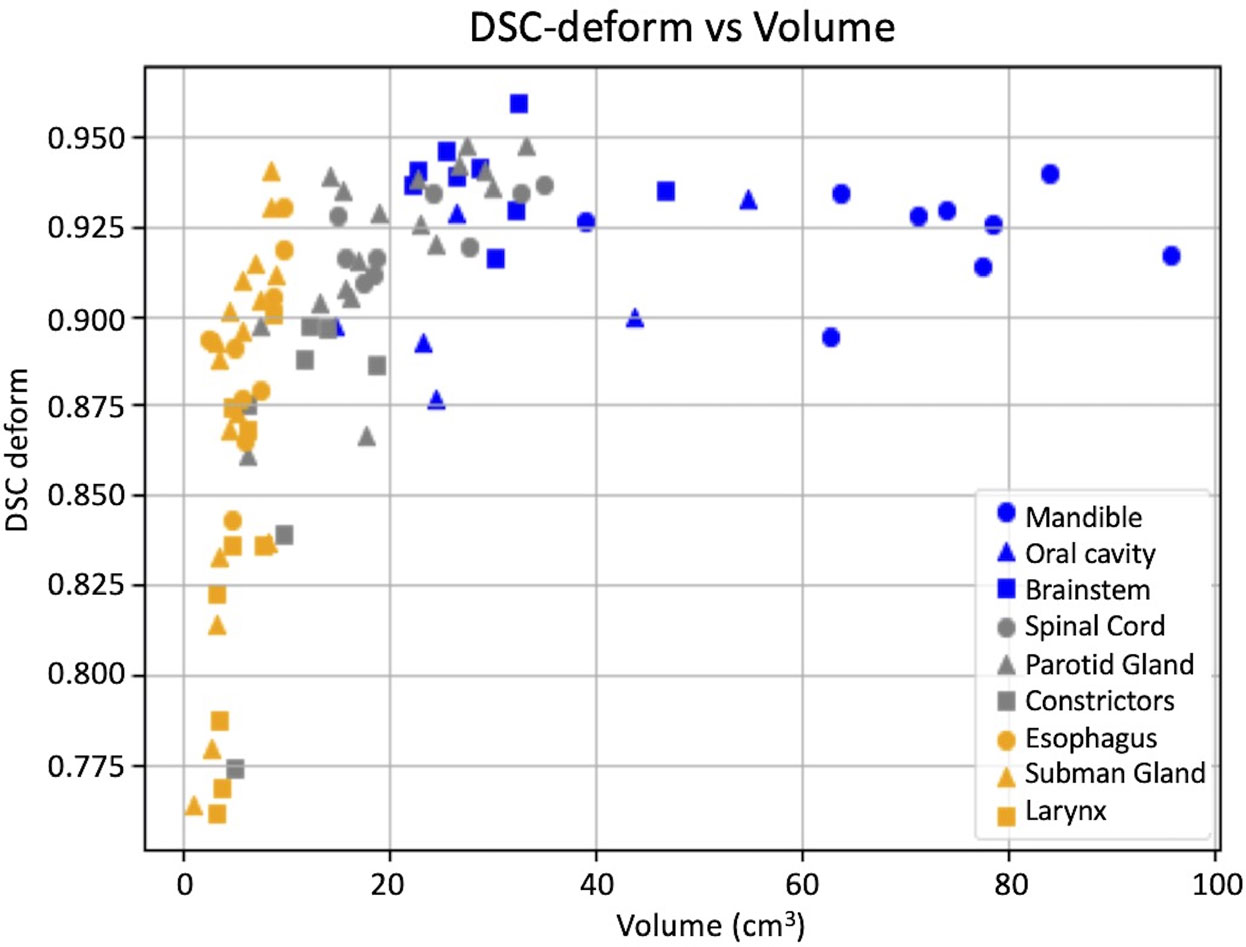
Plot of DSC_DR_ vs contour volume for all kV‐kV contour propagations.

kV‐kV DSC geometric congruence is summarized in Table 3. For each contour group, the table lists the average DSC and SD for both RR and DR contours. On average, the DSC_DR_ scores for both DIR modes, which range from 0.828 to 0.938, are within the TG‐132 recommended range of 0.8 to 0.9. Figure 6 shows the relationship between DSC_RR_ and DSC_DR_ for all contours of interest, again illustrating the improvement in geometric convergence in the process step from RR to DIR. Figure 7 shows the relationship between contour volume and DSC_DR_ for all contours of interest. The variation in DSC is larger for volumes <20 cm^3^ (5.3%) than for volumes >20  cm^3^ (1.8%). This is expected and TG‐132 recommends a DSC congruence range of 0.8 to 0.9, as it may be difficult to achieve higher overlap congruence (DSC) with smaller volumes although surface congruence (MDA) is within voxel dimensions.

**Table 3:** Contour of interest average (±SD) DSC for both RR and DR contour propagation.

Contour	DSC_RR_ Ave + SD	DSC_DR_ Ave + SD
Mandible	0.77 + 0.09	0.92 + 0.01
Oral cavity	0.80 + 0.06	0.90 + 0.02
Brainstem	0.91 + 0.04	0.94 + 0.01
Spinal cord	0.73 + 0.16	0.92 + 0.01
Parotid gland	0.70 + 0.11	0.92 + 0.03
Pharyngeal constrictors	0.66 + 0.19	0.86 + 0.04
Esophagus	0.73 + 0.15	0.89 + 0.03
Submandibular gland	0.64 + 0.20	0.88 + 0.05
Larynx	0.70 + 0.13	0.83 + 0.05

**Fig. 8:**
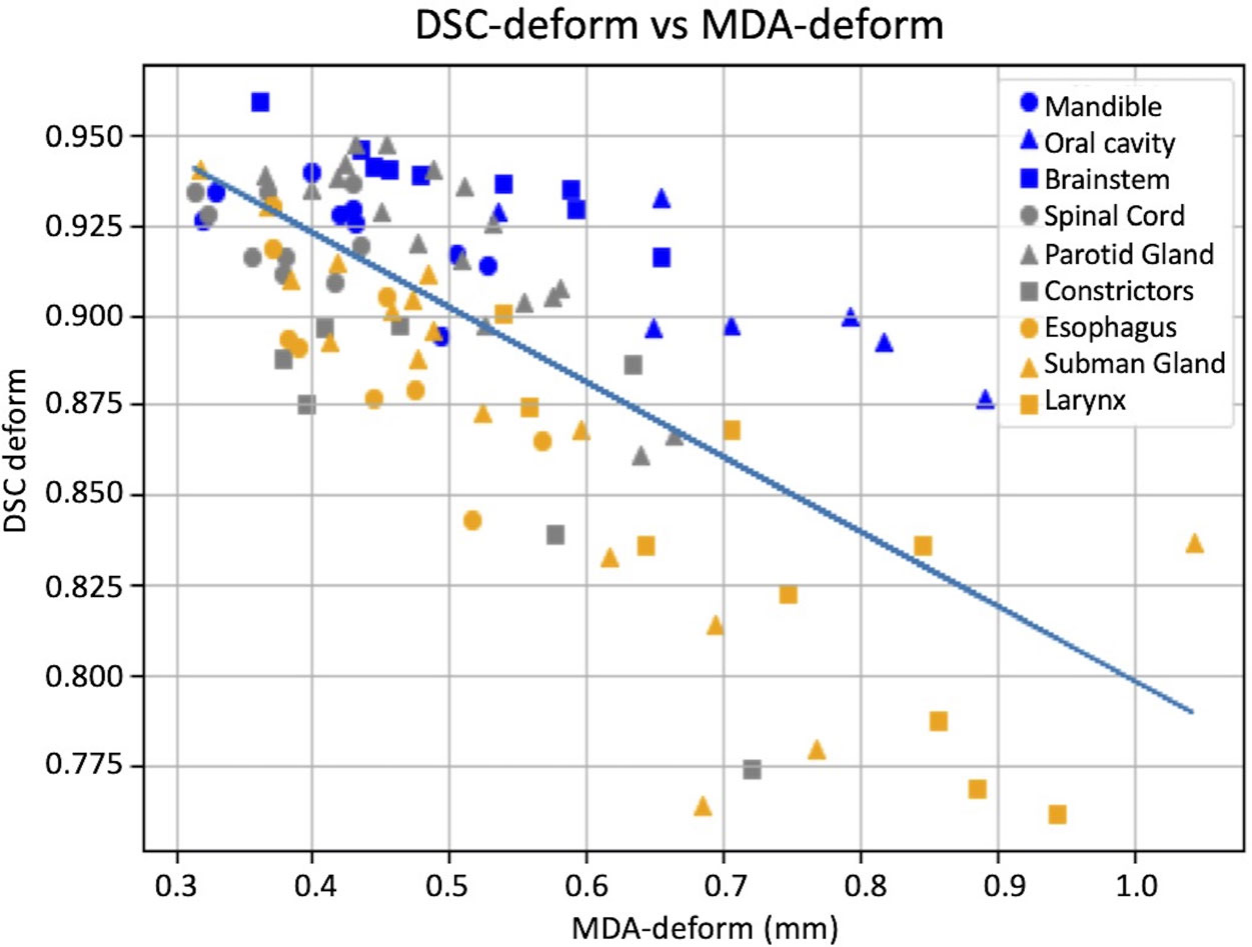
Correlation between DSC_DR_ and MDA_DR_ for all kV‐kV contour propagations. The Pearson correlation coefficient for the plotted regression line is −0.71.

The correlation between MDA_DR_ and DCS_DR_ is plotted in Fig. 8; the Pearson correlation coefficient for the plotted regression line is −0.71. It is notable that the larger volume contours appear predominately above the regression while lower volume contours appear predominately below the regression line. This illustrates again the acceptable range of DSC values recommended by TG‐132 to account for the effects of small volume on contour overlap congruence.

### 3.C kV‐MV contour congruence

kV‐MV MDA geometric congruence in millimeters is summarized in Table 4. For each contour group, the table lists the average and SD MDA for deformable registration (DR) contours. On average, the MDA_DR_ distances are within a voxel width as recommended by TG‐132. Also listed is the Wilcoxon signed‐rank test p‐value to determine if the results are significantly different from kV‐kV DIR results. All contours of interest with the exception of the spinal cord and larynx were within the 0.05 significance level. The oral cavity showed the greatest deviation from kV‐kV DR results.

**Table 4:** Contour of interest average (±SD) MDA in mm for kV‐MV DR contour propagation. Also listed is the Wilcoxon signed‐rank test *P*‐value to determine if the results are significantly different from kV‐kV DIR results.

Contour	MDA_DR_ Ave + SD	Wilcoxon *P*‐value
Mandible	0.4 + 0.1	0.021
Oral cavity	1.0 + 0.5	0.036
Brainstem	0.6 + 0.1	0.008
Spinal cord	0.4 + 0.1	0.139
Parotid gland	0.6 + 0.1	0.002
Pharyngeal constrictors	0.5 + 0.1	0.028
Esophagus	0.5 + 0.1	0.028
Submandibular gland	0.6 + 0.2	0.003
Larynx	0.7 + 0.2	0.767

kV‐MV DSC geometric congruence in millimeters is summarized in Table 5. For each contour group, the table lists the average and SD DSC for deformable registration (DR) contours. On average, the DSC_DR_ distances are within the acceptable range of 0.8–0.9 as recommended by TG‐132. Also listed is the Wilcoxon signed‐rank test p‐value to determine if the results are significantly different from kV‐kV DIR results. All contours of interest with the exception of the oral cavity, spinal cord, and larynx were within the 0.05 significance level.

**Table 5:** Contour of interest average (±SD) DSC for kV‐MV DR contour propagation. Also listed is the Wilcoxon signed‐rank test *P*‐value to determine if the results are significantly different from kV‐kV DIR results.

Contour	DSC_DR_ Ave + SD	Wilcoxon *P*‐value
Mandible	0.92 + 0.01	0.015
Oral cavity	0.88 + 0.05	0.069
Brainstem	0.92 + 0.02	0.008
Spinal cord	0.92 + 0.01	0.110
Parotid gland	0.91 + 0.03	0.003
Pharyngeal constrictors	0.86 + 0.05	0.028
Esophagus	0.88 + 0.02	0.038
Submandibular gland	0.86 + 0.05	0.004
Larynx	0.83 + 0.05	0.594

### 3.D Clinical case results

Tables 6 and 7 show the tabulated results of the autocontour congruency test of the three patients who were re‐simulated midway through treatment and manually re‐contoured for adaptive treatment. For the MDA results (Table 6), 14 of 15 contours had MDA distances within 2 SDs of the mean MDA distance of the contour in the HN phantom dataset, and 10 of 15 contours showed better surface congruency. For the DSC results (Table 7), 14 of 15 contours had DSC values within 2 SDs of the mean DSC value of the contour in the HN phantom dataset, and 11 of 15 contours showed better overlap congruency.

**Table 6:** Tabulated values for MDA (mm) congruency test of clinical patient data compared to the average (+SD) MDA of the HN phantom dataset tests.

	HN dataset Ave + SD	Patient 1	Patient 2	Patient 3
Parotid left	0.5 + 0.1	0.43	0.65	0.61
Parotid right	0.5 + 0.1	0.34	0.69	0.64
Mandible	0.4 + 0.1	0.42	0.34	0.42
Brainstem	0.4 + 0.1	0.25	0.44	0.42
Spinal cord	0.5 + 0.1	0.33	0.38	0.80

**Table 7:** Tabulated values for DSC congruency test of clinical patient data compared to the average (+SD) DSC of the HN phantom dataset tests.

Contour	HN dataset Ave + SD	Patient 1	Patient 2	Patient 3
Parotid left	0.92 + 0.03	0.948	0.922	0.904
Parotid right	0.92 + 0.03	0.958	0.905	0.901
Mandible	0.92 + 0.01	0.930	0.941	0.927
Brainstem	0.94 + 0.01	0.970	0.949	0.953
Spinal cord	0.92 + 0.01	0.934	0.930	0.900

## 4 DISCUSSION

In this study, we have evaluated and validated Accuray DIR contour propagation using a commercially available virtual HN phantom dataset for both kV‐kV and kV‐MV DIR modes. The dataset models the changes in patient anatomy and setup typical for a 30+ fractionated treatment, and therefore is ideal for testing contour propagation for both planning retreatments and adaptive monitoring. Ground‐truth contours had to be generated on the EOT image sets by inverting the DVFs provided with the datasets. Generalized inverse consistency^18^ allowed confidence for inverting the DVFs and propagating the SOT contours to the EOT image sets. Varadhan et al. provide an in‐depth discussion regarding the use of ICE as a framework for the validation of DIR software to provide evidence of a stable system.^6^ However, TG‐132 states that the quantitative metric of inverse consistency does not provide the direct verification of accuracy.

**Fig. 9:**
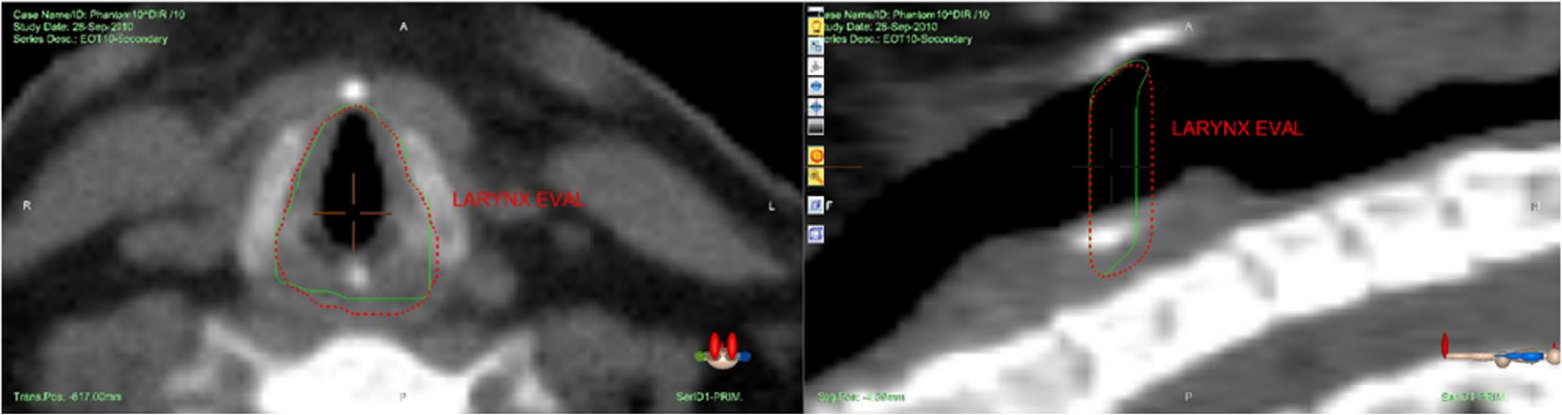
Comparison of ground truth (green) with DR‐transferred (red) larynx contours on the axial and sagittal view of CT image.

Contour geometric congruency metrics, DSC and MDA, were calculated and summarized for each of the nine contours of interest. MDA congruence was within 1‐mm except for one submandibular contour. DSC congruence was within the TG‐132 recommended range of 0.8‐0.9 for 93% of contours. Note that these results are specific to applications involving adaptive treatment management; auto‐segmentation of contours using atlases of different patients may not be as accurate. Contour congruence becomes more difficult as contour volume falls below 20  cm^3^. However, other factors such as initial rigid alignment and change volume size had little effect on DIR accuracy. This is nicely illustrated in the principle component analysis plot shown in Fig. S1. Image slice thickness may have an effect on contour overlap in the inferior and superior extent of the image set as illustrated in Fig. 9. Small volumes that span only a few image slices such as the larynx are especially sensitive to this. The original benchmark study of commercial DIR platforms by Pukala and collegues^9^ analyzed DIR TRE in the source‐target direction from EOT to SOT and did not include the Accuray DIR algorithm at the time. In the interest of comparing the Accuray DIR algorithm with other platforms, we performed a similar study whose results are tabulated in the Table S1. The average TRE_mean_ distances for the brainstem (0.5 mm), spinal cord (0.5 mm), mandible (0.5 mm), and parotid glands (0.5 mm) were in good agreement with an earlier version of MIM (5.6.2); the MIM values were 0.5, 0.5, 0.9, and 1.2/1.5 mm, respectively. Both algorithms use a free‐form deformation model. Although the current study analyzed TRE with DVFs in the opposing source–target direction, the ground‐truth DVFs were inversely consistent for the contour volumes evaluated. Future studies will test dosimetric congruency using the HN dataset and other image datasets.

MVCT‐image quality has a small, but significant effect on autocontour congruence. On the one hand, Murphy et al.^16^ showed no sensitivity to noise when comparing automatic and manual contour variability; they reason that the use of an equivalent cross‐correlation coefficient method for similarity optimization is analogous to using an optimal signal‐to‐noise match filter to detect a known signal in noisy data. Faggiano et al.^19^ demonstrated a DIR algorithm that showed no significant differences between automatic parotid gland contour propagation and manual contouring in HN MVCT images. Whereas, on the other hand, our study did show small but significant differences when comparing intermodal DIR (kV‐MV) using ground‐truth virtual phantoms. Future studies will address contributions from each component of the MVCT image transformation and possible solutions that can be translated into the clinic to improve intermodal DIR. Iterative reconstruction methods available for MVCT imaging on the Accuray Radixact characteristically have better CNR^20^; it is expected that kV‐MV DR agreement is as good or better than the results shown in this study.

## 5 CONCLUSION

The results of this validation study of the Accuray DIR algorithm using a benchmark HN virtual phantom dataset indicate that this DIR algorithm, when applied to adaptive treatment management, can be expected on average to auto‐propagate HN contours adequately within the tolerances recommended by TG‐132. However, contours critical to dosimetric planning should always be visually proofed for accuracy. Standard reconstruction MVCT image quality causes slightly lower, but acceptable, agreement with ground‐truth contours.

## DATA SHARING STATEMENT

The data that support the findings of this study are available from the corresponding author upon reasonable request.

## Supporting information


**Data S1**. **Supplementary Figure 1**: Principal component analysis of registration results. The loadings are the initial rigid alignment congruency results for DSC (Dice r) and MDA (r), the DR congruency results for DCS (Dice d) and MDA (d), the average (ave) and the percent difference (diff) in volume between SOT and EOT. The orthogonality of the rigid alignment and diff loadings to the DR results suggest little to no correlation. The average volume has some positive correlation to DR DSC, and DSC and MDA have negative correlation as would be expected, i.e. the larger the overlap agreement the smaller the mean distance to surface agreement. None of the loadings appear on either principle component axis suggesting little influence in the variation of data, and none of the loadings have component lengths greater than 1 suggesting no particular loading had greater influence on the variationof data.
**Supplementary Table 1:** Comparison of mean registration error statistics for voxels contained in the contour of interest. Statistics are listed as mean ± SD and maximum errors are shown parentheses. Results shown for MIM, Velocity, RayStation, Pinnacle, and Eclipse are from Pukala et al.^9^.
