# Supplementary material for: Validation of accuracy deformable image registration contour propagation using a benchmark virtual HN phantom dataset
Source: J Appl Clin Med Phys. 2021 May 4;22(5):58–68. doi: 10.1002/acm2.13246 (PMC8130232; doi:10.1002/acm2.13246)
Supplement: Supplementary file 1 — Data S1. Supplementary Figure 1: Principal component analysis of registration results. The loadings are the initial rigid alignment congruency results for DSC (Dice r) and MDA (r), the DR congruency results for DCS (Dice d) and MDA (d), the average (ave) and the percent difference (diff) in volume between SOT and EOT. The orthogonality of the rigid alignment and diff loadings to the DR results suggest little to no correlation. The average volume has some positive correlation to DR DSC, and DSC and MDA have negative correlation as would be expected, i.e. the larger the overlap agreement the smaller the mean distance to surface agreement. None of the loadings appear on either principle component axis suggesting little influence in the variation of data, and none of the loadings have component lengths greater than 1 suggesting no particular loading had greater influence on the variationof data. Supplementary Table 1: Comparison of mean registration error statistics for voxels contained in the contour of interest. Statistics are listed as mean ± SD and maximum errors are shown parentheses. Results shown for MIM, Velocity, RayStation, Pinnacle, and Eclipse are from Pukala et al.9. [file ACM2-22-58-s001.pdf]

Supplementary Data

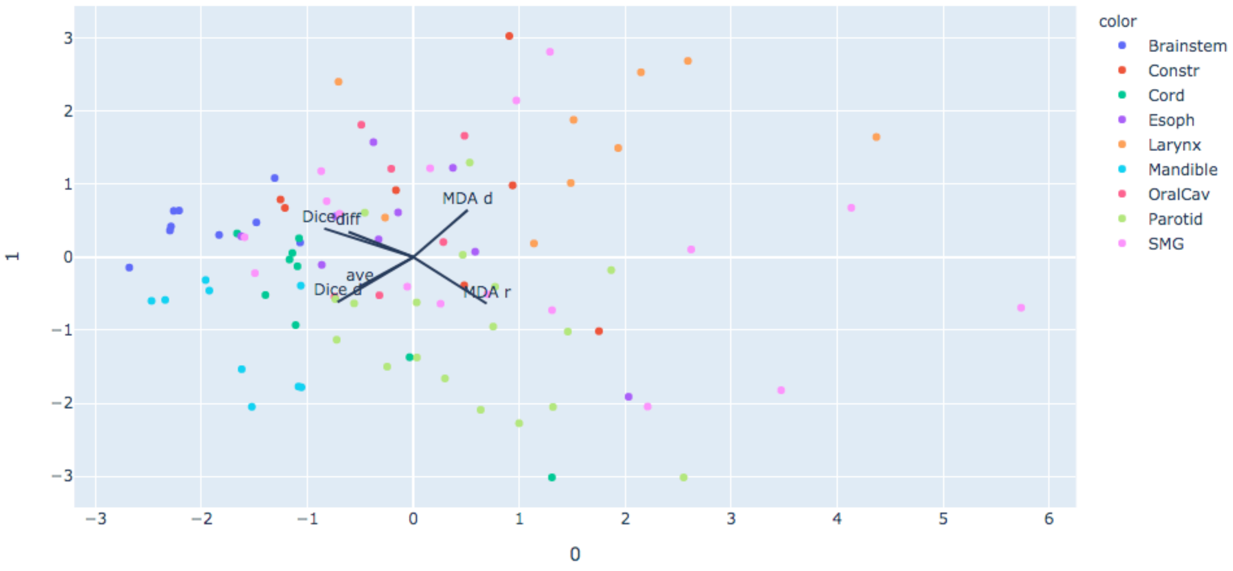

Supplementary Figure 1. Principal component analysis of registration results. The loadings are the initial rigid alignment congruency results for DSC (Dice r) and MDA (r), the DR congruency results for DCS (Dice d) and MDA (d), the average (ave) and the percent difference (diff) in volume between SOT and EOT. The orthogonality of the rigid alignment and diff loadings to the DR results suggest little to no correlation. The average volume has some positive correlation to DR DSC, and DSC and MDA have negative correlation as would be expected, i.e. the larger the overlap agreement the smaller the mean distance to surface agreement. None of the loadings appear on either principle component axis suggesting little influence in the variation of data, and none of the loadings have component lengths greater than 1 suggesting no particular loading had greater influence on the variation of data.

Contour	*Accuray*	*MIM*	*Velocity*	*RayStation*	*Pinnacle*	*Eclipse*
Brainstem	0.5±0.1 (1.6)	0.5±0.2 (2.5)	1.2±0.5 (3.0)	1.4±0.7 (4.1)	3.3±2.1 (16.1)	1.1±0.4 (5.0)
Spinal Cord	0.5±0.1 (1.5)	0.5±0.1 (2.6)	1.8±1.2 (14.8)	1.0±0.8 (7.7)	1.0±0.3 (4.3)	1.1±0.3 (4.2)
Mandible	0.5±0.1 (3.2)	0.9±0.3 (6.3)	1.5±0.4 (5.6)	1.6±0.7 (8.6)	1.2±0.4 (6.4)	2.1±0.9 (9.5)
Lt Parotid	0.5±0.1 (3.2)	1.2±0.6 (10.8)	2.2±0.7 (8.7)	2.0±1.1 (11.8)	1.9±0.7 (9.4)	2.1±0.7 (11.4)
Rt Parotid	0.5±0.1 (1.6)	1.5±1.7 (22.0)	1.6±0.5 (7.9)	2.4±1.6 (15.2)	1.7±0.7 (8.3)	1.8±1.0 (8.5)
External	0.7±0.1 (28.0)	1.4±0.4 (31.5)	1.9±0.3 (23.3)	2.9±0.9 (36.8)	2.7±0.8 (35.0)	2.5±0.3 (25.7)

Supplementary Table 1. Comparison of mean registration error statistics for voxels contained in the contour of interest. Statistics are listed as mean ± SD and maximum errors are shown parentheses. Results shown for MIM, Velocity, RayStation, Pinnacle, and Eclipse are from Pukala et al.[9]
